# Does Vitamin C Deficiency Promote Fatty Liver Disease Development?

**DOI:** 10.3390/nu6125473

**Published:** 2014-12-01

**Authors:** David Højland Ipsen, Pernille Tveden-Nyborg, Jens Lykkesfeldt

**Affiliations:** Department of Veterinary Disease Biology, Faculty of Health and Medical Sciences, University of Copenhagen, Ridebanevej 9, Frederiksberg C, 1870 Copenhagen, Denmark; E-Mails: dhi@sund.ku.dk (D.H.I.); ptn@sund.ku.dk (P.T.-N.)

**Keywords:** antioxidants, obesity, oxidative stress, non-alcoholic fatty liver disease, non-alcoholic steatohepatitis, reactive oxygen species, vitamin C, vitamin C deficiency

## Abstract

Obesity and the subsequent reprogramming of the white adipose tissue are linked to human disease-complexes including metabolic syndrome and concurrent non-alcoholic fatty liver disease (NAFLD) and non-alcoholic steatohepatitis (NASH). The dietary imposed dyslipidemia promotes redox imbalance by the generation of excess levels of reactive oxygen species and induces adipocyte dysfunction and reprogramming, leading to a low grade systemic inflammation and ectopic lipid deposition, e.g., in the liver, hereby promoting a vicious circle in which dietary factors initiate a metabolic change that further exacerbates the negative consequences of an adverse life-style. Large epidemiological studies and findings from controlled *in vivo* animal studies have provided evidence supporting an association between poor vitamin C (VitC) status and propagation of life-style associated diseases. In addition, overweight per se has been shown to result in reduced plasma VitC, and the distribution of body fat in obesity has been shown to have an inverse relationship with VitC plasma levels. Recently, a number of epidemiological studies have indicated a VitC intake below the recommended daily allowance (RDA) in NAFLD-patients, suggesting an association between dietary habits, disease and VitC deficiency. In the general population, VitC deficiency (defined as a plasma concentration below 23 μM) affects around 10% of adults, however, this prevalence is increased by an adverse life-style, deficiency potentially playing a broader role in disease progression in specific subgroups. This review discusses the currently available data from human surveys and experimental models in search of a putative role of VitC deficiency in the development of NAFLD and NASH.

## 1. Introduction

Obesity has become an epidemic with detrimental effects on health and wellbeing. In 2013, more than one-third of the world’s adult population could be characterized as being overweight or obese (body mass index (BMI) ≥25 kg/m^2^) [1]. Obesity has been found to result in dysfunctional adipose tissue, low-grade systemic inflammation and redox imbalance with increased oxidative stress [2,3,4]. As part of the so-called metabolic syndrome, known associations with obesity include a wide range of diseases, e.g., atherosclerosis, insulin resistance (IR), type 2 diabetes and non-alcoholic fatty liver disease (NAFLD) [5]. The development of NAFLD is closely linked to increased levels of circulating lipids, IR, inflammation and oxidative stress [6,7]. In agreement with a putative role of oxidative stress in the etiology of NAFLD, important antioxidant enzymes and vitamins are decreased in obese and NAFLD patients [4,8,9].

Vitamin C (VitC) is a water-soluble, chain-breaking antioxidant capable of scavenging essentially all physiologically relevant free radicals [10]. Additionally, it serves as donor of reducing equivalents in multiple enzymatic reactions, of which its role in proline and lysine hydroxylation during collagen synthesis is probably most widely known [11]. Humans and a few other species are unable to synthesize VitC, and must instead acquire it through dietary means. Inadequate dietary intake results in VitC deficiency defined as plasma levels below 23 μM [12]. Cross-sectional population studies have shown that about 10%–20% of the western population can be diagnosed with VitC deficiency and that poor VitC status is associated with increased all-cause mortality [13,14,15,16,17] Moreover, the prevalence of VitC deficiency in specific subgroups may be considerably higher, potentially enhancing susceptibility to oxidative stress and disease [18]. Accordingly, VitC status correlates inversely with e.g., BMI and is significantly decreased in obese compared with lean individuals [9,19,20,21,22]. Thus, the apparent link between obesity, NAFLD and oxidative stress suggests that NAFLD progression may be accelerated by poor VitC status.

The aim of this review is to discuss the current data from human and animal studies in relation to a putative role of VitC in the development of NAFLD.

## 2. Obesity, Systemic Inflammation and NAFLD

### 2.1. Obesity and Systemic Inflammation

**Figure 1:**
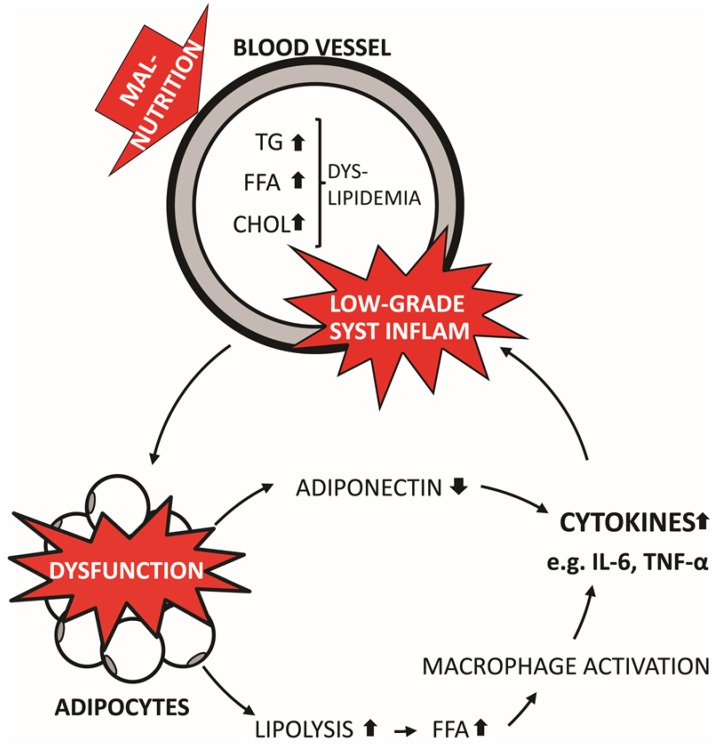
Propagation of low-grade systemic inflammation from adipose tissues.

Obesity is primarily a consequence of a sedentary lifestyle and the intake of an excess amount of calories. As a result, unused resources are accumulated as fat in adipose tissue, facilitating adipocyte hypertrophy and hyperplasia, alongside increased production of chemokines, cytokines, reactive oxygen species (ROS), hypoxia and cell death, ultimately leading to macrophage infiltration and adipocyte dysfunction [23,24,25]. Macrophages initiate and maintain inflammation by secreting a variety of cytokines, such as tumor necrosis factor α (TNFα) and interleukin (IL) 6 [2,23] At the same time, dysfunctional adipocytes increase their secretion of pro-inflammatory cytokines, while decreasing secretion of anti-inflammatory cytokines [24,26]. Decreased secretion of adiponectin by adipocytes and concurrent interference with the insulin signaling pathway by inflammatory cytokines promotes the development of IR [2,27,28]. Furthermore, inflammation of the adipose tissue may participate in the induction of hepatic IR as well [29]. Inflammation, IR and adipocyte hypertrophy per se can increase the rate of lipolysis and subsequent release of free fatty acids (FFA) [2,30,31]. FFAs have been shown to directly activate macrophages through Toll-like receptors 2 and 4 and enhance the production of inflammatory cytokines [32]. Thus, a vicious cycle is established: Adipocyte dysfunction induces a low-grade inflammation, which increases the release of FFA. In turn, the FFAs stimulate macrophage activation and additional production of inflammatory cytokines [5] (Figure 1). Ultimately, this may result in a state of chronic, low-grade systemic inflammation and oxidative stress [33]. Low levels of antioxidants may enhance oxidative stress, aggravating an already vulnerable state of redox imbalance.

Dietary induced dyslipidemia leads to increased fat deposition and promotes hypertrophy and hyperplasia of adipocytes as well as cellular reprogramming leading to altered secretory functions of adipocytes. The tissue expansion and associated adipocyte dysfunction increases the release of inflammatory cytokines (such as IL6 and TNFα) and activates macrophages. Furthermore, release of the anti-inflammatory adipokines, e.g. adiponectin, is decreased. This induces a state of low-grade systemic inflammation, which in turn compromises insulin sensitivity and increases lipolysis in adipose tissues, increasing the release of free fatty acids (FFA) and exacerbating the dietary induced dyslipidemia. Moreover, FFAs also promote macrophage activation by binding to Toll-like receptors 2 and −4, further propagating the production of inflammatory cytokines. Thus a viscous cycle is established in which dyslipidemia and systemic inflammation is propagated through dysfunctions in adipocyte metabolism.

Multiple cross-sectional studies have found decreased levels of VitC and several other antioxidants in obese men and women, and observed an inverse correlation between VitC levels and BMI, waist circumference and body fat percentage [15,19,20,21,22]. The third Glasgow MONICA population survey investigated the plasma VitC status of men and women aged 25–74 years and found 44% of the participants (*n* = 1267) to be VitC deficient, with a VitC plasma concentration below 23 μM [15]. Additionally, using data from 8808 U.S. adult men and women participating in the Third National Health and Nutrition Examination Survey (NHANES III), Ford *et al.* reported that the metabolic syndrome was associated with decreased levels of VitC and other antioxidants such as vitamin E (VitE) [34]. Dietary habits may be an important factor contributing to the poor VitC status in obesity. Obese individuals have been shown to consume less dietary VitC [22]. Similarly, obese women were found to consume less fruits and vegetables compared to lean controls [35].

A putative role of VitC in inflammation has also been suggested, since VitC levels have been found to be inversely associated with inflammatory markers such as C-reactive protein (CRP) and myeloperoxidase [19,22]. Furthermore, VitC may be involved in the regulation of the key adipokine, adiponectin, supported by a recent study reporting dietary VitC intake to correlate with adiponectin levels in adolescent girls [36]. *In vitro* studies have suggested that VitC increases the secretion of high molecular weight (HMW) adiponectin in fully differentiated human adipocytes without changing total adiponectin secretion [37]. Contrary to this, however, supplementation with VitC (500 mg/day) in obese patients did not change adiponectin levels [21]. Quantification of total adiponectin, instead of the bioactive HMW isomer alone, may account for the conflicting results. Alternatively, the dose of VitC might have been too low, as 1000 mg/day was shown to improve several other metabolic parameters in patients with type 2 diabetes, *i.e.*, HbA1c, fasting blood sugar, serum insulin, triglycerides (TG) and low-density lipoprotein (LDL) [38]. Further cell studies have shown that increased oxidative stress decrease the expression of adiponectin mRNA in adipocytes and that adiponectin levels are inversely correlated with 4-hydroxynoneal, a maker of lipid peroxidation [39]. Adiponectin levels were also inversely associated with the severity of hepatic steatosis and necroinflammation [40]. Thus, a role of VitC in adiponectin regulation could have important effects on progression of inflammation, IR and NAFLD.

### 2.2. NAFLD

NAFLD is estimated to affect 6% to 33% (median 20%) of the world’s population and constitutes the most common liver disease in the western world [41,42]. NAFLD comprises a cluster of hepatic conditions ranging from simple, initially benign and reversible steatosis to hepatocyte ballooning, Mallory bodies, fibrosis and inflammation in non-alcoholic steatohepatitis (NASH), [6,43,44,45]. However, in some patients, hepatic steatosis progresses to NASH, increasing the risk of hepatic cirrhosis and hepatocellular carcinoma [43,46]. NAFLD is strongly associated with obesity, dyslipidemia, IR, oxidative stress, inflammation and the metabolic syndrome [47,48]. Indeed, the prevalence of NAFLD in obese patients has been reported to be 74%, the amount of visceral adipose tissue in particular being highly correlated with increased risk of NAFLD [6,49]. The pathogenesis of NAFLD is not yet completely understood. However, it is currently believed that initial hepatic fat accumulation is followed by multiple parallel hits that promote inflammation and disease progression. The most important events believed to be involved in this process include; IR, increased FFA, dietary factors, cytokines derived from the adipose tissue and ROS formation [7].

IR is a key mechanism in hepatic dyslipidemia. Expectedly, diabetic patients have an increased prevalence of NAFLD compared to non-diabetic individuals, supporting the link between IR and NAFLD [50]. While IR increases circulating levels of FFAs, hyperinsulinemia during IR, paradoxically, apparently inhibits hepatic lipid oxidation and increases *de novo* lipogenesis [51]. Concurrently, saturation of very-low-density lipoprotein (VLDL) secretion may facilitate lipid accumulation as well [52]. In patients with NAFLD, the majority of hepatic lipids originate from circulating FFA [53]. FFA from visceral adipose tissue can drain directly into the portal vein and be transported straight to the liver. As hepatocytic FFA uptake is mediated by passive diffusion, increased FFA levels result in increased FFA uptake, which is subsequently stored as TG [6,54]. Interestingly, a meta-analysis of clinical trials investigating the effect of VitC intervention on LDL, high-density lipoprotein (HDL) and TGs in patients with hypercholesterolemia suggested that VitC supplementation can reduce the concentration of lipids in circulation (TG and LDL-C) [55]. Additionally, adipose tissue-derived cytokines may contribute to the development of NAFLD, and inflammation of the adipose tissue may indeed precede hepatic inflammation [56,57,58]. Adiponectin has been shown to reduce ectopic hepatic lipid accumulation, systemic IR and inflammation [59]. In addition, TNFα and IL6 have received much attention as putative key cytokines involved in hepatic lipid accumulation, inflammation and IR [60].

**Figure 2:**
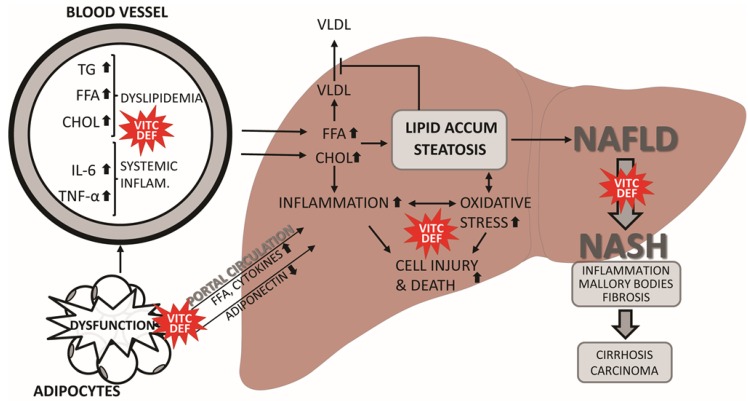
Putative effects of vitamin C deficiency on the progression of NAFLD.

Dietary factors also play an important role in NAFLD. High dietary fructose, trans-fatty acids, and cholesterol have all been found to contribute to the development of dyslipidemia, hepatic fat accumulation and subsequent disease progression [7,61,62,63,64,65]. FFA and cholesterol not only induce hepatic lipid deposition, but may also have injurious effects in the liver, in part by inducing hepatocyte apoptosis, lipotoxicity, mitochondrial dysfunction and endoplasmic reticulum stress with subsequent ROS formation and inflammation [24,66,67,68,69,70,71]. Furthermore, activation of hepatic stellate cells and Kupffer cells, e.g., by hepatocyte apoptosis and ROS, also appear to promote disease progression through the secretion of TNFα, IL6, transforming growth factor β and collagen, which induces inflammation and fibrosis [72] (Figure 2). Indeed, increased generation of ROS and lipid peroxidation are believed to be key pathogenic components in NAFLD, perpetuating inflammation, fibrogenesis, and development of cirrhosis and hepatocellular carcinoma [73,74]. Oxidative stress alone has been found to be associated with NAFLD both in patients with, but also without type 2 diabetes [75]. VitC has been shown to decrease mitochondrial ROS formation and stimulate the activity of manganese superoxide dismutase (SOD) and glutathione peroxidase (GPx) in isolated rat liver mitochondria [76]. However, dietary intake of VitC and VitE has been reported to be lower in NAFLD patients, potentially predisposing them to oxidative stress [77,78].

As co-factor for the 7α-hydroxylase, catalyzing the conversion of cholesterol to 7α-hydroxycholesterol, VitC deficiency may increase circulating levels of cholesterol (CHOL) by reducing its excretion through the bile. Systemic inflammation and dyslipidemia is propagated through adipocyte dysfunction, e.g., releasing fatty acids (FFA) and inflammatory cytokines to the blood stream. Moreover, by affecting adiponectin regulation, lack of VitC may further increase dyslipidemia, systemic inflammation and oxidative stress. As VitC is also a powerful antioxidant, deficiency per se promotes cellular oxidative stress, e.g., in the liver. The excess lipids induce mitochondrial dysfunction and stress of the endoplasmic reticulum (ER) further propagating hepatic oxidative stress and inflammation. FFA and CHOL is taken up by hepatocytes and processed to be exported from the liver by VLDL. When overloaded, hepatic VLDL export is saturated and lipids are accumulated in hepatocytes. Ultimately, NAFLD progression is driven by increased oxidative stress and inflammation, promoting activation of hepatic stellate and Kupffer cells, which alongside an increased hepatic production of TNFα, IL6, transforming growth factor β and collagen, amplifies the induction of cellular damage and fibrosis. Combined with key pathogenic changes taking place during the advancement from simple steatosis (NAFLD) to steatohepatitis (NASH), e.g., hepatocyte ballooning, formation of Mallory bodies and fibrosis, NASH may progress even further to hepatic cirrhosis and hepatocellular carcinoma.

Consistent with this notion, a number of investigators have found that markers of oxidative damage such as malondialdehyde (MDA) and protein carbonyls are elevated, while antioxidants like catalase and SOD are decreased in the plasma and livers of animals and humans with NAFLD [8,65,79,80]. This has led to the hypothesis that VitC therapy could prove beneficial in the treatment of NAFLD (Figure 2).

## 3. Does Vitamin C Deficiency Promote NAFLD?

### 3.1. Animal Studies of Vitamin C Deficiency

Several *in vivo* studies have investigated the role of poor VitC status in relation to dyslipidemia and NAFLD (Table 1). Like humans, guinea pigs are unable to synthesize VitC due to a non-functional gene encoding for l-gulonolactone oxidase (gulo), making them an excellent model for the study of VitC deficiency. Moreover, with regards to, e.g., cholesterol distribution, activity of enzymes involved in lipoprotein metabolism, LDL receptor binding domain characteristics and cholesterol synthesis, guinea pigs are comparable to humans in contrast to other rodents [81]. Contrary to the guinea pig, the gulo gene is functional in mice and rats and both species are able to synthesize VitC. However, the effect of VitC deficiency can be examined in genetically engineered rodent models, although the validity and human relevance of such models may be difficult to assess.

Guinea pigs kept on a diet without VitC for 4 weeks showed elevated levels of hepatic cholesterol and TG compared to control animals on a VitC supplemented diet [82]. Similarly, another study in guinea pigs showed increased hepatic triacylglycerol and cholesteryl esters after 6 weeks of VitC deficiency [83]. Scurvy can be avoided by supplementing the diet with small amounts of VitC, allowing investigators to study the long-term effects of non-scorbutic VitC deficiency. After up to 31 weeks on such a diet, guinea pigs had elevated levels of TG in the liver, thoracic aorta and serum. This was due to decreased conversion of cholesterol to bile acid, revealing a crucial role of VitC in this process [84]. Specifically, VitC is a co-factor for the 7α-hydroxylase catalyzing the conversion of cholesterol to 7α-hydroxycholesterol, constituting the rate-limiting step in bile acid formation, and thus, VitC deficiency results in reduced excretion of cholesterol in animals [85]. The addition of cholesterol to a VitC deficient diet exacerbated the effects of VitC deficiency per se by increasing the amount of hepatic TG, cholesterol, focal fibrosis and proliferation of connective tissue. Moreover, high dose (100 mg/day, p.o.) of VitC significantly diminished these changes [86]. Interestingly, dietary cholesterol also appears to have effects on the VitC status. In a long-term dietary intervention study in guinea pigs, high cholesterol fed animals consistently showed 33% (range 27%–43%) lower plasma and liver VitC concentrations compared to control animals regardless of low or high dietary VitC intake [87]. In the osteogenic disorder shionogi (ODS) rat—a mutant rat strain unable to synthesize VitC—VitC deficiency with or without cholesterol and cholic acid supplementation for 19 days increased hepatic cholesterol and total lipids [88]. Furthermore, regardless of the amount of cholesterol added to the diet, Uchida *et al.* did not find any effect of VitC relating to changes in hepatic lipids in ODS rats [89]. These discrepancies may reflect the significant differences between guinea pigs and rats in the way lipids are handled and stored [90,91]. Collectively, the above results suggest that VitC is involved in the regulation of both circulating and hepatic lipid homeostasis, supporting VitC as an important factor in the development of NAFLD.

**Table 1:** Animal studies of vitamin C deficiency.

Species	Age/Weight	Design	Outcome	Ref.
Guinea Pigs	200–300 g	Control (VitC 5 mg VitC/day) Deficient (0.5 mg VitC/day) High VitC (100 mg VitC/day) ± 100 mg chol/day Duration: 16 weeks	VitC deficiency, liver: TG ↑ Chol ↑ PL ↔ VitC deficiency + chol, liver: TG ↑ Chol ↑ PL ↔ Histology reveal fat infiltration, local necrosis and proliferation of connective tissue	[86]
Guinea Pigs	400–450 g	Control (1000 mg VitC/kg diet) VitC deficient (0 mg VitC/kg diet) VitC high (25,000 mg VitC/kg) Duration: 4 weeks	VitC deficiency, liver: Chol ↑ TG ↑ VitC deficiency, serum: Chol ↑ TG ↑ HDL ↓ LDL ↑ VLDL ↓	[82]
Guinea Pigs	300–450 g	Control (10 mg VitC/day) Deficient (0.5 mg VitC/day) Duration: 22, 28 or 31 weeks	VitC deficiency, liver/serum/thoracic aorta: Chol ↑	[84]
Guinea Pigs	?	Control (660 mg VitC/kg diet) Deficiency (33 mg VitC/kg diet) High (13,200 mg VitC/kg diet) Duration: 5 weeks	VitC deficiency, liver: TBARS ↑ MDA ↑ protein carbonyls ↑	[92]
Guinea Pigs	250–300 g	Control (25 mg VitC/kg/day) Deficiency (0 mg VitC/kg/day) Duration: 21 days	VitC deficiency, liver: TBARS ↑	[93]
Guinea Pigs	600–700 g	Control (500 mg VitC/kg diet) Deficiency (50 mg VitC/kg diet) Duration: 6 weeks	VitC deficiency, liver: TAG ↑ CE ↑ FC ↔	[83]
Guinea Pigs	12 weeks	High or low fat diet with different VitC: Low (100 mg VitC/kg diet) High (691 mg VitC/kg diet) Duration: 6 months	High fat diet, liver: VitC ↓	[87]
Mice, SMP30^−/−^ and WT	30 days	SMP30^−/−^ or WT ±1.5 mg/L VitC in water Duration: 57 days	VitC deficiency, liver: protein carbonyl ↑ SOD-activity ↑ Cu Zn-SOD protein expression ↑ TBARS ↔, CAT protein expression ↓	[94]
Mice, Gulo^−/−^ or WT	20–21 weeks	16 weeks on western diet, then: Control (0.33 g VitC/L in drinking water) Deficiency (0 g VitC/L in drinking water) Duration: 3 weeks	VitC deficiency: GSH ↔ MDA ↑ F2- an F4-isoprostanes ↔	[95]
Mice, Gulo^−/−^	Newborn	Control Gulo^+/+^ (0 mg VitC) Deficiency Gulo^−/−^ (0 mg VitC) Duration: 18 days	Gulo^−/−^, liver: MDA ↑ protein carbonyl ↑ sulfhydryls ↔ GSH ↑	[96]
Rats, ODS or WT	6 weeks	ODS fed 0, 50, 300, 3000 mg VitC/kg diet ± 0.5% chol and 0.25% cholic acid WT fed 0 mg VitC/kg diet ± 0.5% chol and 0.25% cholic acid Duration: 19 days	VitC deficiency, liver: Chol ↑/↔ total lipids ↑/↔ VitC deficiency, serum: Chol ↔ HDL-C ↔ VitC deficiency + chol/cholic acid, liver: Chol ↑, total lipids ↔ VitC deficiency + chol/cholic acid, serum: Chol ↑, HDL-C ↑	[88]
Rats, ODS	6 weeks	Control 300 mg VitC/kg diet Deficient 0 mg VitC/kg diet Duration: 14 days	VitC deficiency, liver: CINC-1 ↑ Apo-A1 mRNA ↓ ApoE mRNA ↔	[97]
Rats, ODS or WT	9 weeks	Control (30 mg VitC/L in drinking water ± 0.5% chol Deficiency 0 mg/L in drinking water Duration: 3 weeks	VitC deficiency, liver: Chol ↔ TG ↔ PL ↔ VitC deficiency, serum: Chol ↑ Total lipoprotein ↓ HDL ↓IDL ↓ LDL ↑ VLDL ↓ VitC deficiency + chol did not affect serum levels further. Chol feeding, regardless of VitC status, increase hepatic lipids	[89]

CAT: Catalase. Chol: Cholesterol. CE: cholesteryl ester. CINC-1: Cytokine-induced neutrophil chemoattractant-1. FC: Free cholesterol. GSH: Glutathione. Gulo: l-gulonolactone oxidase HDL: High-density lipoprotein. IDL: Intermediate-density lipoprotein. LDL: Low-density lipoprotein. MDA: Malondialdehyde. ODS: Osteogenic Disorder Shionogi. PL: Phospholipids. SMP30: Senescence marker protein. SOD: Superoxide dismutase. TAG: Triacylglycerol. TBARS: Thiobarbituric acid reactive substances. TG: Triglyceride. VitC: Vitamin C. VLDL: Very-low-density lipoprotein. WT: Wild type; Ref.: Reference; ↓ Decrease; ↑ Increase; ↔ No change.

In accordance with a central role in the prevention of oxidative stress, VitC deficiency has been shown to cause a reduction in both expression and activity of various antioxidant enzymes with a concurrent rise in markers of oxidative stress. Thus, 3–5 weeks of VitC deficiency caused increased levels of thiobarbituric acid reactive substance (TBARS), MDA and protein carbonyls in the liver of guinea pigs [92,93]. Oxidative stress was also evident in the liver of gulo^−/−^ mice without access to VitC for 57 days. Protein carbonyls, but not TBARS, were increased in the livers of these animals, alongside decreased catalase protein expression compared to WT or gulo^−/−^ supplemented controls [94]. However, SOD activity was increased, likely as a compensatory mechanism [94]. Indeed, SOD appears to play an important role in NAFLD as increased hepatic fibrosis is found in NASH patients with mutations in the gene coding for SOD2 [98]. Pierce *et al.* found increased hepatic MDA in VitC deficient gulo^−/−^ mice placed on a western diet, but with no change in glutathione or isoprostanes [95]. However, other authors report an increase of glutathione a long with increased MDA and protein carbonyls in livers of gulo^−/−^ mice deprived of VitC for similar periods of time [96]. These differences between studies may be attributed to different diet compositions and age of the animals, *i.e.*, a western type diet and 4–5 weeks old animals *vs.* normal chow and newly born animals, respectively. The considerable number of studies reporting increased protein carbonyls, MDA and TBARS in VitC deficient animals, support that VitC has a protective effect against hepatic protein and lipid oxidation. This may be of significance as lipid oxidation has been shown to occur in NAFLD and even correlate with the severity of liver necroinflammation and fibrosis [99]. Furthermore, keeping ODS rats on a VitC free diet for 14 days resulted in increased cytokine-induced neutrophil chemoattractant-1 (CINC-1), a potent inflammatory chemokine, in serum, liver and spleen. Thus, VitC deficiency may mediate the infiltration and accumulation of neutrophils with subsequent inflammatory damage in some tissues [97].

### 3.2. Animal Studies of VitC Intervention and NAFLD

Methionine and choline are essential for hepatic β-oxidation and VLDL production. Expectedly, methionine and choline deficient (MCD) diets cause accumulation of intra-hepatic lipids and decreased VLDL synthesis [43]. Rezazadeh and coworkers fed rats a MCD diet for 10 weeks, and randomized animals to receive VitC or no supplementation for an additional eight weeks on the diet [100]. While VitC supplementation significantly decreased hepatocellular ballooning and inflammation, liver steatosis still persisted. At the same time, hepatic markers of oxidative stress were decreased, while SOD and catalase were increased [100]. Similar results were obtained by supplementing rats placed on a MCD diet with VitC for 10 weeks. The authors also found that VitC treatment significantly increased glutathione reductase (GR) and GPx activity [101]. Moreover, VitC treatment resulted in a significant decrease in circulating liver enzymes, TG and LDL, showing a protective effect of VitC supplementation on the MCD diet-induced dyslipidemia [100,101]. Conversely, rats fed a choline deficient diet for 4 weeks with concurrent VitC supplementation did not show any improvements in serum TG or aspartate aminotransferase, even though hepatic steatosis was ameliorated and oxidative stress reduced [102]. However, the studies differ considerably in diet composition, duration and rat strain used, confounding compatibility between findings.

Imbalance of the antioxidant status, similar to that observed in the animal studies, has also been found in patients with steatosis while antioxidant status is even further compromised in NASH [80]. Animal models utilizing MCD or choline deficient diets to induce NAFLD appear to present a similar pathology, but the pathogenesis is different from the human situation. Additionally, most animals lose weight on the MCD diet, contrary to the human situation, in which many NAFLD patients are obese. Results may therefore not be directly translatable.

Guinea pigs placed on an atherogenic diet, high in fat and cholesterol, for 4 months developed dyslipidemia with elevated serum cholesterol and phospholipids in addition to elevated hepatic levels of cholesterol, phospholipids and TG [103]. VitC deficiency exacerbated the dyslipidemia and hepatic consequences, but high VitC reduced the severity of both dyslipidemia and hepatic lipid accumulation [103]. It is noteworthy, that extremely high doses of VitC (13,200 mg/kg diet) have been shown to decrease hepatic markers of oxidation to the same degree as normal VitC doses in guinea pigs, but also cause a reduction of body weight and GR activity, similarly to what was observed in the VitC deficient animals [92]. It is possible that the very high dose of VitC caused adverse effects in the guinea pigs, e.g., gastrointestinal disturbances and osmotic diarrhea [104,105], which could affect bodyweight and general physiology, however, this was not reported by the authors. Conversely, Roomi *et al.* did not report any adverse effects of a mega-dose of VitC (25,000 mg VitC/kg diet) in guinea pigs [82]. Contrary to guinea pigs, supplementation with high levels of VitC in ODS rats did not reduce hepatic cholesterol to a greater extent that normal VitC levels did [88]. Thus, VitC treatment seemingly reduces hepatic oxidative stress, but have variable effect on hepatic steatosis, likely depending on the employed method of induction, *i.e.*, a high fat diet *versus* a MCD or MD diet (Table 2).

### 3.3. Epidemiological Studies of VitC Intake and NAFLD

A number of cohort studies have been carried out, investigating the dietary habits of patients with NAFLD. Results are conflicting; some studies indicate suboptimal VitC intake by NAFLD patients while others do not (Table 3). Using a 7-day food record Musso *et al.* evaluated dietary habits in patients with NASH and healthy controls. Among other differences, NASH patients consumed significantly less VitC compared to the healthy controls (84.3 ± 43.1 *vs.* 144.2 ± 63.1 mg) [77]. Males constituted the vast majority of the NASH group (24/25 patients) and though below that of healthy controls, dietary intakes were close to the recommended daily intake of 90 mg VitC/day, although this was not the case for smokers with NASH [77,106]. Contrary to this, Ferolla and colleagues found that intake of VitC in NAFLD patients diagnosed with fatty liver, did not meet recommendations when assessed in men and women using 24-h dietary recall and food frequency questionnaires (FFQ) [78]. Unfortunately, the lack of a control group impedes investigation as to whether low VitC intake was also prevalent in a comparable healthy population. Different methodology, ethnicity and patients’ disease stage may account for apparent study dissimilarities. Indeed, food records may themselves lead patients to change their dietary patterns [107].

**Table 2:** Animal studies of VitC intervention in NAFLD.

Species	Age/Weight	Design	Outcome	Ref.
Guinea Pigs	300 g	Normal or atherogenic diet Control (10 mg/kg/day) Deficiency (1 mg/kg/day) High (25 mg/kg/day) VitC administration: Oral Duration: 4 months	Compared to control (normal diet): VitC deficiency, liver: TC ↑ TG ↑ PL ↓ serum: TC ↔ TG ↔ PL ↔ High VitC, liver: TC ↔ TG ↓ PL ↑ serum: TC ↔ TG ↔ PL ↔ Compared to control (atherogenic diet): VitC deficiency, liver: TC ↑ TG ↑ PL ↓ serum: TC ↑ TG ↑ PL ↔ High VitC, liver: TC ↓TG ↔ PL ↓ serum: TC ↓ TG ↓ PL ↓	[103]
Rats	250–300 g	Control (MCD diet for 10 weeks, then 8 additional weeks of MCD diet + vehicle) Treatment (MCD diet for 10 weeks, then 8 additional weeks of MCD diet + VitC 30 mg/kg/day) VitC administration: Oral Duration: 18 weeks	VitC, liver: ballooning ↓ inflammation ↓ steatosis ↔ SOD ↑ CAT ↑ protein carbonyls ↓ VitC, serum: AST ↓ ALT ↓ ALP ↓ γGT ↓ TC ↓ HDL ↑ LDL ↓	[100]
Rats	250–300 g	Control (MCD diet + vehicle) Treatment (MCD diet + VitC 30 mg/kg/day) VitC administration: Oral Duration: 10 weeks	VitC, liver: ballooning ↓ SOD ↑ CAT ↑ GR ↑ GPx ↑ TBARS ↓ protein carbonyls ↓ VitC, serum: AST ↓ ALT ↓ ALP ↓ γGT ↓ TC ↓ HDL ↑ LDL ↓	[101]
Rats	300–350 g	Control (CD diet + vehicle) Treatment (CD diet +30 mg VitC/kg/day) VitC administration: Oral Duration: 4 weeks	VitC, liver: prevents steatosis and reduces oxidative stress VitC, plasma: AST ↔ TG ↔	[102]

ALP: Alkaline phosphatase; ALT: Alanine aminotransferase; AST: Aspartate aminotransferase; CAT: Catalase; CD: Choline deficient; GPx: Glutathione peroxidase; GR: Glutathione reductase; γGT: Gamma-glutamyl transferase; HDL: High-density lipoprotein; LDL: Low-density lipoprotein; MCD: methionine choline deficient; PL: Phospholipids; SOD: Superoxide dismutase; TBARS: Thiobarbituric acid reactive substances; TC: Total cholesterol; TG: Triglyceride; VitC: Vitamin C; Ref.: Reference; ↓ Decrease; ↑ Increase; ↔ No change.

Han and co-workers studied the dietary intake of Korean adults [108] and reported no differences in dietary VitC intake between men and women with NAFLD and control subjects However, when adjusted for age, job, education level, exercise frequency, smoking status, energy intake and *n*–3 fatty acid intake, VitC intake was negatively associated with the risk of NAFLD in male patients (OR: 4.23; *p*-trend = 0.014) [108]. In children with steatosis or NASH, Vos *et al.* found dietary intakes of VitC to be similar [109]. However, while differences in steatosis grade were not associated with differences in VitC intake, significantly increased hepatocyte ballooning was indeed observed in children with decreased VitC consumption [109]. In another study, Mager *et al.* reported that daily recommendations of VitC were met by children with NAFLD [110]. However, impaired antioxidant status was implied as measured by low levels of red blood cell-glutathione compared to previous reports in healthy children although the conclusion is weakened by the lack of paired controls [110]. Da Silva and associates concluded that VitC intake was not different between healthy controls and adults with either steatosis or NASH [111]. In this study of 74 NASH patients and 27 healthy controls, the intake correlated with median VitC plasma concentrations, which did not differ between groups 67.6 and 72.1 μM, respectively [111]. Likewise, Madan *et al.* did not find any differences in VitC plasma levels between 29 NAFLD patients and 23 healthy controls [112]. Also, the VitC plasma concentrations were found not to correlate with hepatic inflammation or fibrosis [112]. However, in another study by Cabakan and co-workers, 105 NASH patients had reduced VitC plasma levels alongside increased plasma MDA and nitric oxide compared with steatosis patients [113]. It is possible that this reflects an ability of VitC to slow down the progression of NAFLD.

The conflicting results do not allow a definitive conclusion regarding dietary VitC intake. However, the NAFLD patients included in the studies mentioned above largely seem to have met recommended daily allowance of VitC. Unfortunately, most of the studies only examine the estimated dietary intake of VitC and have not actually measured plasma VitC concentrations. It has been suggested that assessments of dietary VitC only have a moderate relationship with plasma VitC levels, thus the two measures may not describe the VitC status in the same way [114]. Moreover, dietary questionnaires have certain limitations and underreporting is known to occur, which may also skew results [107]. As noted previously, oxidative stress is increased in NAFLD patients [75]. As such, they may have an increased demand of antioxidants, like VitC, and inadequate levels are not likely to be reflected by reports of dietary intake alone. Daily doses of VitC between 30 and 100 mg do not result in plasma saturation, leading some investigators to propose that the daily recommended dose of VitC be increased from 75 and 90 mg/day (US women and men, respectively) [105], to 200 mg/day in healthy individuals [115]. Furthermore, small differences in dietary intake of VitC between 30 and 100 mg/day can lead to vastly different plasma concentrations, as this interval represents a very steep part of the plasma concentration *vs.* dose curve [115]. Therefore, patients with a daily VitC intake in this area and even corresponding to the current recommendation may still be at risk of having plasma VitC concentrations below optimal levels. While they may not strictly be VitC deficient, plasma VitC levels between 23 and 50 μM may still leave patients vulnerable to disease, as, e.g., the relative risk of heart failure has been shown to increase significantly by every 20 μM drop in VitC plasma concentrations [116].

**Table 3:** Epidemiological studies of VitC status in NAFLD patients.

Design	Groups	Outcome	Ref.
Cross-Sectional	Adults Healthy controls (*n* = 25) NASH (*n* = 25)	NASH patients consumes less dietary VitC (*p* = 0.0001)	[77]
Cross-Sectional	Adults NAFLD patients (*n* = 96)	Dietary VitC intake was below recommended levels	[78]
Cross-Sectional	Adults Male healthy controls (*n* = 63) Male NAFLD (*n* = 103) Women healthy controls (*n* = 116) Women NAFLD (*n* = 66)	Dietary intake of VitC was not different in men and women with NAFLD compared with control (*p* = 0.666) Intake of VitC correlated negatively with the odds-ratio of NAFLD for male patients (OR: 4.23, *p*-trend = 0.014)	[108]
Cross-Sectional	Children Steatosis (*n* = 39) Borderline Z3 (*n* = 27) Borderline Z1 (*n* = 36) NASH (*n* = 47)	Dietary VitC intake was similar in all groups (*p* = 0.15) and above recommended levels High grade of steatosis was not associated with lower dietary VitC intake (*p* = 0.97) Amount of hepatocyte ballooning increases with lower dietary VitC levels (*p* = 0.05)	[109]
Cross-Sectional	Children NAFLD (*n* = 38)	Dietary VitC intake was in agreement with recommended levels	[110]
Cross-Sectional	Adults Healthy control (*n* = 27) Steatosis (*n* = 33) NASH (*n* = 41)	Plasma concentrations of VitC did not differ between groups (*p* > 0.05) Dietary intake of VitC did not differ between groups (*p* > 0.05)	[111]
Prospective	Adults Healthy controls (*n* = 23) NAFLD (*n* = 29)	Plasma VitC concentrations were not different between groups (*p* = 0.65) Plasma VitC concentrations did not correlate with inflammatory grade (*p* = 0.56) or fibrosis stage (*p* = 0.53)	[112]
Cross-Sectional	Adults Fatty liver disease (*n* = 38) NASH (*n* = 67)	Plasma VitC concentrations were lower in NASH patients (*p* = 0.001)	[113]

NAFLD: Non-alcoholic fatty liver disease; NASH: Non-alcoholic steatohepatitis; OR: Odds ratio; VitC: Vitamin C; Ref.: Reference.

### 3.4. Clinical Intervention Studies with VitC and NAFLD

To date, no study has directly examined the effect of VitC treatment alone in NAFLD and compared it to a placebo-treated control group. However, studies which combine VitC with other treatment form(s) have been conducted (Table 4). A 12 month, double-blinded, randomized controlled trial (RCT) examined the differences between placebo and combined treatment with VitC (500 mg/day) and E (600 IU/day) in children [117]. Both groups received lifestyle intervention, involving increased physical activity and a tailored diet aimed at inducing weight loss or maintaining weight in overweight and normal weight children, respectively. Liver brightness (as a measure of lipid accumulation), examined by ultrasound, circulating levels of liver enzymes, cholesterol and TG were reduced in both groups compared to baseline, but were not significantly different between groups [117]. Subsequently, the study was continued as an open-label study for an additional 12 months, but with fewer participants and the inclusion of a post-treatment liver biopsy [118]. Compared to baseline, both groups exhibited significant improvements in steatosis grade, lobular inflammation, hepatocyte ballooning and liver enzymes, but not in fibrosis scores. However, again there were no differences between placebo and treatment group. Thus, VitC and E supplementation are seemingly not superior to lifestyle intervention in children with NAFLD [118]. However, due to the study design, it is not possible to determine if vitamin treatment is better than no treatment at all. The authors rightfully suggested that increased fruit and vegetable consumption, augmented by the new diet, may well have minimized the effect of the vitamin treatment [118]. Indeed, VitC absorption is highly dose depended and excess amounts are effectively excreted and can therefore not facilitate additional health benefits [115]. Harrison *et al.* examined the effect of VitC (1000 mg/day) in combination with VitE (1000 IU/day) *versus* placebo after 6 months treatment of NASH patients [119]. Lifestyle interventions in both groups focused on exercise and a low-calorie diet. Hepatic inflammation, necrosis, fibrosis and liver enzymes were not significantly different between the groups. However, a significant, albeit clinically modest, intra-group improvement was observed in the vitamin treated group, with regards to hepatic fibrosis. On the basis of this result, the authors concluded that VitC and VitE treatment seemingly improves hepatic fibrosis in NASH patients [119]. However, this conclusion has been criticized; emphasizing that valid conclusion in placebo-controlled trials must be made by comparing treatment and placebo-groups rather than with historic data [120]. Interestingly, the fibrosis score was not improved in children with NAFLD, when compared to baseline [118]. Both studies implemented lifestyle changes, but doses of VitC and E, study duration and participants were different between the two studies. Additionally, the children with NAFLD had a low fibrosis score (0–1) at baseline, which may impede detection of potential improvements [118]. Importantly, liver physiology, development and the features of NASH may differ considerably between children and adults, which could affect study outcomes [121,122].

**Table 4:** Clinical intervention studies with VitC in NAFLD.

Design	Groups and Intervention		Outcome	Ref.
12 months, double-blinded, randomized clinical trial	Children Lifestyle intervention + placebo (*n* = 43) Lifestyle intervention + VitC (500 mg/day) and VitE (600 IU/day) (*n* = 45)		No differences between groups (*p* > 0.05)	[117]
24 months 12 month double-blinded followed by 12 month open-label, randomized clinical trial	Children Lifestyle intervention + placebo (*n* = 28) Lifestyle intervention + VitC (500 mg/day) and VitE (600 IU/day) (*n* = 25)		No differences between placebo and VitE/VitC groups (*p* > 0.05) Compared to baseline, treatment improved steatosis grade (*p* < 0.001), lobular inflammation (*p* < 0.001), hepatocyte ballooning (*p* < 0.001) and NAFLD activity score (*p* > 0.001), but not portal inflammation (*p* = 0.1) and fibrosis stage (*p* = 0.6).	[118]
6 month, double-blinded, randomized clinical trial	Adults Lifestyle intervention + placebo (*n* = 22) Lifestyle intervention + VitC (1000 mg/day) and VitE (1000 IU/day) (*n* = 23)		No differences between placebo and VitC/VitE groups (*p* > 0.05) Compared to baseline, VitC/VitE treatment improves fibrosis (*p* = 0.002)	[119]
12 months, pilot study No control group	Adults VitC (300 mg/day) and VitE (300 mg/day (*n* = 23)		Treatment decreased serum ALT (*p* < 0.0001) and hs-CRP (*p* < 0.005) and improved steatosis (6/10), necroinflammation (8/10), fibrosis (4/10).	[123]
6 month, open-label, randomized study	Adults Ursodeoxycholic acid (10 mg/kg/day) (*n* = 29) VitC (500 mg/day) and VitE (600 IU/day) (*n* = 27)		No differences between ursodeoxycholic acid and VitC/VitE treatment (*p* > 0.05)	[124]
4 years, randomized clinical trial	Adults NAFLD Placebo (*n* = 36) Treatment (1000 mg VitC/day, 1000 IU VitE/day, 20 mg atorvastatin/day) (*n* = 44)	Adults Normal liver Placebo (*n* = 190) Treatment (1000 mg VitC/day, 1000 IU VitE/day, 20 mg atorvastatin/day (*n* = 185)	Treatment reduced risk of having moderate to severe hepatic steatosis (OR = 0.36, *p* < 0.017)	[125]

ALT: Alanine aminotransferase; Hs-CRP: High sensitivity C-reactive protein; NAFLD: Non-alcoholic fatty liver disease; OR: Odds ratio; VitC: Vitamin C; VitE: Vitamin E; Ref.: Reference.

In a pilot study, NASH patients were treated with VitC (300 mg/day) and VitE (300 mg/day) for 12 months and changes were compared to baseline. BMI remained unchanged, but serum alanine aminotransferase and high-sensitivity-CRP were significantly reduced by the antioxidant treatment [123]. High-sensitivity-CRP has been shown to be associated with the severity of liver pathology in NASH [126]. Ten of the 23 study participants underwent liver biopsy post-treatment and of these, steatosis improved in 6/10, necroinflammation in 8/10 and fibrosis in 4/10 [123]. However, the lack of a placebo group confounds these findings with time-induced changes and complicates the interpretation of the potential effect of the combined VitC and E treatment. The effect of ursodeoxycholic acid (UDCA) (10 mg/kg/day) compared to that of VitC (500 mg/day) and VitE (600 IU/day) was examined in a 6 month, open-label, randomized study [124]. Compared to baseline, both treatments decreased circulating levels of liver enzymes without inducing changes in liver echogenicity. Side effects were reported in the UDCA treated group, whereas no side effects were reported in the vitamin treated group [124]. This is in agreement with other studies reporting that VitC and E are well tolerated [119,123]. Foster and coworkers evaluated the combination of atorvastatin (20 mg/day), VitC (1000 mg/day) and VitE (1000 IU/day) [125]. CT-scans were used to identify NAFLD. Subsequently, the participants were divided into NAFLD patients and normal liver patients. These two groups were further allocated to either the treatment combination or placebo. The four groups were reexamined after 2 and 4 years. In patients without NALFD at baseline, the treatment had no preventive effect. However, in patients with NAFLD, active treatment significantly reduced the prevalence of fatty liver [125]. This is contrary to the result of Ersöz *et al.*, who found no improvement in hepatic echogenicity after 6 months of treatment [124]. However, considerably lower vitamin doses, duration and different radiological methods were used by Ersöz *et al.*, which may explain the conflicting results. However, improvements in hepatic steatosis may of cause, in part be contributed to atorvastatin, as hepatic steatosis is associated with dyslipidemia [127]. Indeed, active treatment with VitC, VitE and atorvastatin did reduce plasma levels of LDL and cholesterol, while HDL and TG levels remained unchanged [125]. Furthermore, treatment with atorvastatin (20 mg/day) alone, improved ultrasound echo-patterns in NAFLD patients [128]. Unfortunately, radiological techniques are not able to detect small amounts of hepatic steatosis and can therefore not distinguish between the different forms of NAFLD [129]. Consequently, the effect of the treatment on more advanced stages of NAFLD is unknown.

## 4. Conclusion

Controlled animal experiments support a role of VitC deficiency in the elevation of plasma and hepatic lipids, alongside increased hepatic oxidative stress, fibrosis and inflammation. In agreement, VitC treatment of liver disease-induced animals has been shown to reduce hepatic markers of oxidative stress. Clinical studies of the relationship between VitC deficiency and NAFLD have not been conducted and studies of estimated dietary intakes of VitC in NAFLD patients are conflicting. Some studies show correlations between VitC intake and NAFLD progression, while others do not. Importantly, only a few studies have addressed VitC plasma levels in NAFLD patients [111,112,113]. The presently available RCTs have not found an effect of VitC superior to that of placebo. However, a number of studies do demonstrate hepatic improvements compared with baseline, but the use of treatment-cocktails and concurrent life-style interventions seriously confound the analysis of VitC specific effects. Additionally, most RCTs have not recorded or reported baseline VitC concentrations and putative effects of VitC supplementation cannot be assessed in patients already saturated with VitC due to its non-linear absorption kinetics [16]. Therefore, the role of VitC in NAFLD should be investigated in future RCTs in which plasma VitC status is controlled for.
